# New data in France on the trematode *Alaria alata* (Goeze, 1792) obtained during *Trichinella* inspections

**DOI:** 10.1051/parasite/2011183271

**Published:** 2011-08-15

**Authors:** J. Portier, D. Jouet, H. Ferté, O. Gibout, A. Heckmann, P. Boireau, I. Vallée

**Affiliations:** 1 JE 2533 – USC Anses “VECPAR”, UFR de Pharmacie, Université de Reims Champagne-Ardenne 51, rue Cognacq-Jay 51096 Reims France; 2 Anses, Laboratory for Animal Health, Anses Maisons-Alfort, UMR BIPAR, ENVA, UPEC 23, avenue du Général de Gaulle 94706 Maisons-Alfort France; 3 Laboratoire départemental d’Analyses vétérinaires et alimentaires Chemin des Champs de la Loge BP 216 10006 Troyes Cedex France

**Keywords:** trematode, *Alaria alata*, wild boar, *Sus scrofa*, *Trichinella*, resistance, trématode, *Alaria alata*, sanglier, *Sus scrofa*, *Trichinella*, résistance

## Abstract

The trematode *Alaria alata* is a cosmopolite parasite found in red foxes (*Vulpes vulpes*), the main definitive host in Europe. In contrast only few data are reported in wild boars (*Sus scrofa*), a paratenic host. The aim of this paper is to describe the importance and distribution of *Alaria alata* mesocercariae in wild boars, information is given by findings of these larvae during *Trichinella* mandatory meat inspection on wild boars’ carcasses aimed for human consumption. More than a hundred cases of mesocercariae positive animals are found every year in the East of France. First investigations on the parasite’s resistance to deep-freezing in meat are presented in this work.

## Abstract (fr)

*Alaria alata* (Diplostomidae : Trematoda) est parasite à l’état adulte de l’intestin grêle du Renard roux (*Vulpes vulpes*), le principal hôte définitif en Europe. Les données existantes sur ce parasite chez le Sanglier (*Sus scrofa*), le principal hôte paraténique, sont fragmentaires. L’objectif de cet article est de préciser l’importance et la distribution d’*Alaria alata* chez les sangliers en France grâce aux déclarations de mésocercarioses obtenues lors des recherches obligatoires de Trichine sur les carcasses ces dernières années. Le recueil de ces déclarations montre que plus de 100 cas d’animaux positifs sont signalés chaque année, principalement dans le nordest de la France. Une première approche de la résistance à la congélation d’*Alaria alata* dans la viande est présentée dans ce travail.

## Introduction

The presence of *Alaria alata* has been described in wild boars and pigs (*Sus scrofa*) since the late 19^th^ century (Duncker, 1896). In comparison with definitive hosts, few data on *A. alata* in European Suidae are reported (Jakšic *et al.*, 2002, Grösse & Wüsste, 2006, Milešević *et al.*, 2004) and according to the fluke’s life cycle, they are considered as paratenic hosts (Dollfus & Chabaud, 1953). The life cycle of *A. alata* is complex and involves several hosts and tissue locations according to the parasite stages. Indeed, the adults of *A. alata* (Diplostomidae, Trematoda) are intestinal parasites of canids [Red Fox (*Vulpes vulpes*), Wolf (*Canis lupus*), Raccoon Dog (*Nyctereutes procyonides*), Dog (*Canis lupus familiaris*)], the definitive hosts. After hatching of the eggs, the miracidia penetrate the first intermediate host (freshwater snails) to evolve after two generations of sporocysts who produce the furcocercariae (Ruszkowski, 1922). The second intermediate host, an amphibian, is infected by this stage and gives *in situ*, the mesocercaria. Usually, the definitive host is infected by predation. The mesocercariae migrate to the lungs and evolve to the metacercarial stage which migrates back to the intestines (the larvae migrate up the pulmonary tract and are swallowed) and finally develops to the mature adult stage. Some modifications are observed according to the species. The transmammary infections by mesocercariae are possible with *Alaria marcianae* where adults serve as paratenic hosts and offspring might serve as definitive hosts (Shoop *et al.*, 1990). *Alaria mustelae* needs four hosts: the metacercarial stage does not develop to the adult stage in the same host (Bosma, 1931).

Isolations of *A. alata* mesocercariae have occurred in France during meat inspections for *Trichinella* spp. in wild boars’ meat aimed for human consumption. The analysis for the zoonotic nematode *Trichinella* is indeed compulsory in pigs and wild boars and relies in the European Union on an EU regulation (Regulation EC 2075/2005). During these inspections, a new report of *Alaria* sp. mesocercariae was made in December 2004 in the Aube department (North East of France). These parasites were confirmed as mesocercariae of *A. alata* (Goeze, 1792). Since the description of Chabaud *et al*. (1953), there was no report on *A. alata* in France, but since its re-emergence in 2004, it is regularly identified in some area from the East of France.

Within the genus *Alaria*, the species of the new world are considered as zoonotic with seven published cases of human alariosis, all from American or Canadian patients supposedly infested with these parasites (Möhl *et al.*, 2009). One case was lethal: a young Canadian died of a massive infestation, which caused multiple haemorrhages, mainly in the lungs (Fernandez *et al.*, 1976). This young man supposedly ate insufficiently cooked frogs legs. In other cases, larvae were found under the skin (nodule), in the eye or in the respiratory tract. The very little knowledge available on the species *A. alata* must lead to an analysis of the risk for human consumption. Only one experiment showed effects of massive experimental infestation on a primate (Odening, 1963). During this experimentation, several hundreds of *A. alata* mesocercariae were given to a Rhesus monkey, which developed severe clinical signs but did not die. Autopsy showed a generalized alariosis. No human case of alariosis has been reported in Europe and regarding more specifically *A. alata*, there is no available data concerning its potential danger given by human consumption. However, it is evident that a zoonotic risk can be questioned and that the potential danger regarding food safety of wild boars’ meats has to be taken into consideration. In this context, the aim of the present work was to analyse the distribution of *A. alata* in French wild boars and presents preliminary tests on its cryo-resistance.

## Material and Methods

### Artificial digestion of meat

Wild boars meat was treated first for *Trichinella* inspection according to the method described in the EU regulation 2075/2005. Briefly, the artificial digestion relies on a chlorhydropepsic digestion of muscle, which allows the release of free larvae. In France, all routine laboratories performing *Trichinella* meat inspection use the method with magnetic stirrer (Vallée *et al.*, 2007), which is considered as the reference method for *Trichinella* meat inspection (EU Regulation 2075, 2005). Each carcass is analysed with at least 5 g of meat from tongue, diaphragm or foreleg in a pooled sample weighting 100 g.

Samples are digested during 30 min in a beaker containing 2 L water, 10 g powdered pepsin and 16 ml 25% chlorhydric acid. The digestion fluid is passed through a 180 μm mesh sized sieve and decanted in a glass funnel for 30 min. One hundred ml are then harvested in a graduated cylinder and decanted for 10 min. The top 90 ml are eliminated smoothly, and the bottom 10 ml are poured in a Petri dish for *Trichinella* larvae search. Observations are made using a stereomicroscope (× 40 magnification).

### Isolation of *Alaria* mesocercaria

Parasitic elements corresponding morphological criteria (Dollfus & Chabaud, 1953) for *Alaria* mesocercariae observed during Wild boars’ meat digestion are all sent from laboratories to the national reference laboratory at the ANSES Maisons-Alfort for confirmation.

### Molecular identification of *Alaria* developmental stages

DNA was extracted from mesocercariae from wild boars and adult worms isolated from the intestine of red foxes (*Vulpes vulpes*) using the Qiamp DNA mini kit (Qiagen, Germany) following manufacturer’s instructions. Sequencing of the D2 domain and the second internal transcribed spacer (ITS-2) of the 28S unit of the ribosomal DNA were used for the identification. PCR was performed in a 50 μl volume using 5 μl of DNA, and 50 pmol of each of the primers. The PCR mix contained (final concentrations) 10 mM Tris HCl (pH 8.3), 1.5 mM MgCl2, 50 mM KCl, 0.01% Triton X 100, 200 μM dNTP each base, and 1.25 units of Taq polymerase (Eppendorf, Germany). The D2 domain was amplified using the primers used by Mollaret *et al*. (1997): C2’B and D2. ITS-2 was amplified using primers ITS3Trem and ITS4Trem previously described by Dvořák *et al*. (2002). The initial denaturation at 94 °C for 3 min was followed by 40 cycles of denaturation at 94 °C for 45 s, annealing at 50 °C for 45 s and extension at 68 °C for 2 min with a final elongation time of 10 min at 68 °C. PCR products were directly sequenced in both directions with the primers used for DNA amplification (QIAGEN, Germany). The sequences were deposited in GenBank under accession numbers JF340217 to JF340229. Sequence alignment was performed using the ClustalW routine included in the MEGA version 3.1 software (Kumar *et al.*, 2004) and checked by eye and comparison with those deposited in Genbank.

### Cryo-resistance tests

Six positive carcasses were analysed for tests on cryo-resistance. Samples were taken from diaphragm, coastal muscles and legs with a final weight of 100 g per condition. They were tested before freezing and after 2, 3, 5, 10, 11, 18 and 19 days freezing (- 18 °C ± 2 °C). Temperature was monitored with an automatic recording system during all the freezing process with a control every 2 min. After freezing, meat was defrosted at room temperature and treated by artificial digestion for larvae releasing as previously described. Mesocercariae were then analysed under a stereomicroscope (× 40 magnification) to evaluate their mobility.

## Results

### Identification of mesocercariae

The morphology of the trematode larvae showed high similarity with *A. alata* mesocercariae described by Chabaud & Dollfus (1953). Regarding molecular identification, all sequences of the D2 domain of the mesocercariae (n = 1) the adult (n = 2) and the egg (n = 2,) tested were similar and 100% homologous with the sequences of *A. alata* deposited in Genbank (accession number AF184263), from an adult fluke isolated from Raccoon Dog (*Nyctereutes procyonoides*) (Tkach *et al.*, 2001). No variations were observed for the ITS-2 domain between the nine samples sequenced: mesocercariae (n = 6), and eggs (n = 2).

### Geographical distribution of *A. alata* positives wild boars’ carcasses

**Fig 1.**
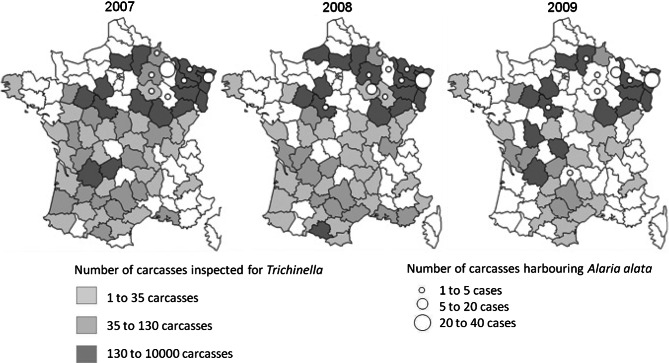
Geographical distribution of mesocercariosis cases and intensity of *Trichinella* inspection on wild boars’ carcasses.

The presence of *A. alata* was detected for the first time in 2004 from a pool of wild boars in Aube department. From 2007 to 2009, the number of infected carcasses identified increases from 62 to 94 in eight departments from East of France. Excepted in this area, other cases were observed in Loir-et-Cher in Centre and Cantal in Middle South respectively in 2008 and 2009 (Fig. 1).

### Cryo-resistance tests

*A. alata* mesocercariae were found alive in fresh meat and in meat frozen at - 18 °C ± 2 °C for two and five days. Most larvae identified were mobile at least five days post-freezing, however it was not possible to evaluate survival after five days since no mesocercariae were found in the meat at days 10 to 19 (Table I).

**Table I.** Analysis of the cryo-resistance of *Alaria alata* mesocercariae in muscle samples from six positive wild boars’ carcasses.

Freezing duration (days)	Carcass identity	Mass analysed per carcass (± 2 g)	Total number of larvae	Number of mobile larvae
0	A	100 g	2	2
	B	100 g	1	1
	C	100 g	1	0
	D	100 g	9	9
	E	200 g	4	4
	F	100 g	2	2

2	A	100 g	5	1
	B	100 g	0	/
	C	100 g	0	/

3	A	100 g	0	/

5	D	3 × 100 g	1	1
	E	100 g	0	/
	F	100 g	0	/

10	D	3 × 100 g	0	/

11	E	3 × 100 g	0	/
	F	3 × 100 g	0	/

18	E	3 × 100 g	0	/

19	E	3 × 100 g	0	/

## Discussion

This work provides an updating on the presence of *A. alata* in French wild boars’ populations since the last report published by Dollfus & Chabaud (1953). The identification of *A. alata* joins in the context of the compulsory diagnosis for trichinellosis and have indeed benefits of the Quality Assurance plan developed in France since 2004 to increase the sensitivity of *Trichinella* detection in meat (Vallée *et al.*, 2007). Since this first description in 2004, the fluke has been found in 11 French departments mainly in East of France. During these three last years, *A. alata* did not expand widely throughout the French territory. However, *Trichinella* inspection has also enabled detection of *Alaria* larvae in wild boars’ meat in other countries like Croatia (Milešević, 2004) or Germany (Grosse and Wüste, 2006).

Although *A. alata* larvae have been identified during *Trichinella* meat inspection, the chlorhydro-pepsic digestion is meant to detect *Trichinella* sp. larvae and not *Alaria* mesocercariae. *A. alata* seems to behave as a *larva migrans* in wild boars and does not appear as encysted (direct observations at the laboratory) although other descriptions show cysts (Leuckart, 1901). The digestion method used for *Trichinella* inspection may dissolve mesocercariae, leading to false negative results (Riehn *et al.*, 2010). Sieves used for this method have 180 μm mesh size, whereas fresh larvae are 460 to 680 μm length and 118 to 338 μm width (Brumpt, 1945; Dollfus & Chabaud, 1953). In these conditions, *Alaria* larvae may not all pass through the sieve. Chlorhydro-pepsic digestion as defined by European regulation CE 2075/2005 may only permit detection of *A. alata* mesocercariae in highly infested meat samples. Recent works have shown that other methods may be more efficient than the artificial digestion method designed for *Trichinella* (Riehn *et al.*, 2010). Moreover, wild boars meat samples used for *Trichinella* inspections are the muscles from the tongue, the masseter or the diaphragm. Digestion must be performed only on muscle tissues and not on fat tissues. Very little information is available on the distribution of *Alaria* mesocercariae in the wild boars’ carcasses and mesocercariae distribution may not match *Trichinella* distribution. More studies are thus needed to analyse the distribution of mesocercariae in paratenic hosts but this would involve experimental infections of wild animals, which are difficult to envisage. However such work would allow to target some tissues to increase the sensitivity of the detection.

The parasite *A. alata* is thus present in a high number of wild boars carcasses but very little is known about the risk for human consumption. The cryo-resistance tests described in this work have shown that larvae can resist at least five days in frozen meat. Unfortunately, the absence of larvae after five days’ freezing did not allow to assess the survival of larvae after this duration. Cryo-resistance tests have not shown a limit of mesocercariae resistance to freezing. The best method of killing these larvae is still cooking: 71 °C remains the recommended temperature to inactivate *Trichinella* larvae (Gamble *et al.*, 2000). This is the reason why the same treatment should be used for *A. alata* inactivation.

The compulsory inspection for *Trichinella* in wild boars has allowed to show the importance of the distribution of *A. alata* in France. This parasite has been present for a long time and its distribution is probably larger than showed in this work. Although no human case has been described in Europe, *A. alata* may be able to cause illness as the American species of *Alaria* did. This potential risk for food safety must be taken into account and the resistance of this parasite to freezing treatments as well as its distribution in wild boar carcasses remain to be evaluated. More sensitive detection methods should also be investigated, which include the choice of the tissues to be targeted for analysis as well as the method of detection.
